# The SNARE protein FolVam7 mediates intracellular trafficking to regulate conidiogenesis and pathogenicity in *Fusarium oxysporum* f. sp. *lycopersici*

**DOI:** 10.1111/1462-2920.14585

**Published:** 2019-03-20

**Authors:** Bing Li, Ying Gao, Hui‐Ying Mao, Katherine A. Borkovich, Shou‐Qiang Ouyang

**Affiliations:** ^1^ College of Horticulture and Plant Protection Yangzhou University Yangzhou China; ^2^ Key Laboratory for Plant‐Microbe Interaction Fujian Agriculture and Forestry University Fuzhou China; ^3^ Department of Microbiology and Plant Pathology, Institute for Integrative Genome Biology University of California Riverside CA USA; ^4^ Joint International Research Laboratory of Agriculture and Agri‐Product Safety of Ministry of Education of China Yangzhou University Yangzhou China; ^5^ Key Laboratory of Plant Functional Genomics of the Ministry of Education Yangzhou University Yangzhou China

## Abstract

Soluble N‐ethylmaleimide‐sensitive factor attachment protein receptors (SNAREs) are conserved in fungi, plants and animals. The Vam7 gene encodes a v‐SNARE protein that involved in vesicle trafficking in fungi. Here, we identified and characterized the function of FolVam7, a homologue of the yeast SNARE protein Vam7p in *Fusarium oxysporum* f. sp. *lycopersici* (*Fol*), a fungal pathogen of tomato. FolVam7 contains SNARE and PX (Phox homology) domains that are indispensable for normal localization and function of FolVam7. Targeted gene deletion showed that FolVam7‐mediated vesicle trafficking is important for vegetative growth, asexual development, conidial morphology and plant infection. Further cytological examinations revealed that FolVam7 is localized to vesicles and vacuole membranes in the hyphae stage. Moreover, the Δ*Folvam7* mutant is insensitive to salt and osmotic stresses and hypersensitive to cell wall stressors. Taken together, our results suggested that FolVam7‐mediated vesicle trafficking promotes vegetative growth, conidiogenesis and pathogenicity of *Fol*.

## Introduction

In eukaryotic cells, directional protein transport between different organelles/compartments of the endomembrane system is mediated by vesicle trafficking and is essential for the survival of organisms (Kienle *et al*., 2009). Vesicle trafficking between diverse organelles is carried out by multiprotein complexes consisting of protein families that have been conserved throughout eukaryotic evolution (Bonifacino and Glick, 2004; Jahn and Scheller, 2006). Soluble N‐ethylmaleimide‐sensitive factor attachment protein receptor (SNARE) proteins are part of the core machineries of these different membrane fusion events and have been characterized extensively in mammals, plants, *Saccharomyces cerevisiae* and phytopathogens (Burri *et al*., 2003; Burri and Lithgow, 2004; Kienle *et al*., 2009; Song *et al*., 2010; Dou *et al*., 2011; Qi *et al*., 2016; Zhang *et al*., 2016; Li *et al*., 2017). With the availability of genomic sequences, a few SNARE proteins have been identified in various species, including 36 in humans, 20 in *Drosophila melanogaster*, 62 in *Arabidopsis thaliana*, 21 in *Aspergillus oryzae* and 24 in *S. cerevisiae* (Pelham *et al*., 1999; Sanderfoot *et al*., 2000; Gupta and Heath, 2002; Burri *et al*., 2003, Burri and Lithgow, 2004; Kuratsu *et al*., 2007). However, only a small number of SNARE proteins have been characterized in plant pathogenic fungi, such as MoSyn8, MoTlg2, MoVam7 and MoSec22 in the rice blast fungus *Magnaporthe oryzae* (Song *et al*., 2010; Dou *et al*., 2011; Zuo *et al*., 2014; Qi *et al*., 2016), UmYup1 in the corn smut fungus *Ustilago maydis*, and FgVam7 and GzSyn1/2 in *Fusarium graminearum*, where they play important roles during development and virulence by mediating vesicle trafficking in the pathogens (Wedlich‐Soldner *et al*., 2000; Hong *et al*., 2010; Zhang *et al*., 2016). Additionally, the t‐SNARE MoSso1 is also involved in virulence, as it is required for the formation of a normal biotrophic interfacial complex (BIC) and for the secretion of cytoplasmic effectors during *M. oryze* infection (Giraldo *et al*., 2013).

SNAREs are a family of conserved proteins involved in intracellular membrane trafficking from one cellular compartment to another. SNARE proteins share a conserved structure, the SNARE domain, which consists of 60–70 amino acids arranged in heptad repeats (Sutton *et al*., 1998; Pratelli *et al*., 2004). SNAREs are functionally classified into v‐SNAREs and t‐SNAREs, according to vesicle related and target membrane related respectively. SNAREs are also divided into four subfamilies, Qa‐, Qb‐, Qc‐ and R‐SNAREs, based on sequence similarity of their SNARE domains and by the presence of conserved glutamine (Q) or arginine (R) amino acid residues in the central portion of the domain (Fasshauer *et al*., 1998; Ungar and Hughson, 2003). In the model of SNARE‐mediated membrane fusion, SNARE proteins localize in the opposing membrane by releasing free energy during the formation of a four‐helix bundle. The formation of this bundle leads to a tight connection between membranes that are destined to fuse and initiate the membrane merger. Conversely, the recycling of SNAREs is achieved through the dissociation of the helical bundle mediated by the N‐ethylmaleimide‐sensitive factor (NSF) (Jahn and Scheller, 2006).

*Fusarium oxysporum* is a worldwide occurring, soil‐borne vascular fungal pathogen causing root rot or wilting disease on a wide variety of plants such as banana, cotton, potato, tomato, capsicum and melons (Michielse and Rep, 2009; Fisher *et al*., 2012). Each *F. oxysporum* strain has been classified into forma specialis (f. sp.) based on its host range (Armstrong and Armstrong *et al*., 1981). For example, tomato (*Solanum lycopersicum*) is the main host of *F. oxysporum f. sp. lycopersici, or Fol* (Kashiwa *et al*., 2016). The infection process of *Fol* has been well studied in tomato, where it displays apparent gene‐for‐gene relationships (Michielse and Rep, 2009). *Fol* invades roots and subsequently colonizes the xylem vessels, thereby compromising water transport and resulting in wilting of the plant (Michielse and Rep, 2009).

Because SNARE family proteins are highly conserved among fungi, study of these proteins may shed light on the roles of exocytosis and endocytosis during pathogenesis in *Fol*. In this study, we identified and functionally characterized the role of the *S. cerevisiae* v‐SNARE Vam7 homologue FolVam7 in *Fol*. We found that FolVam7 is important for vegetative growth, asexual development and pathogenesis *of F. oxysporum* f. sp. *lycopersici*. Our results suggest that the FolVam7 protein may be a novel target for fungicides.

## Results

### *Identification of the FolVAM7 gene and generation of a ΔFolvam7 mutant strain*

Previous studies have shown that fungal SNARE proteins homologous to Vam7 play an important role in fungal development and vacuole fusion (Wedlich‐Soldner *et al*., 2000; Dou *et al*., 2011; Zhang *et al*., 2016). We identified an orthologue of Vam7 (FOXG_02319) in the *F. oxysporum* f. sp. *lycopersici* genome (http://fungidb.org/fungidb/) by a BLAST_P search using the Vam7 protein sequence from *S. cerevisiae*. FOXG_02319 encodes a polypeptide of 362 amino acids, which we named FolVam7. Domain prediction tools revealed that FolVam7 contains a PHOX homology motif (PX) (6–116 residues) at the N‐terminus and a SNARE domain (302–360 residues) at the C‐terminus (http://smartemblheidelberg.de/) (Supporting Information Fig. S1B). FolVam7 shares a variable degree of similarity with other fungal Vam7 proteins: 25.6% with *S. cerevisiae*, 51.6% with *M. oryzae* and 40.59% with *U. maydis* (Supporting Information Fig. S1A).

The high amino acid sequence similarity and shared domain composition prompted us to speculate that FolVam7 might possess conserved biological roles in *Fol*. To test this hypothesis, we generated the knockout mutant Δ*Folvam7* (Supporting Information Fig. S2A). Hygromycin‐resistant transformants were screened by PCR and verified by Southern blot hybridization. Southern blot analysis revealed that the *FolVAM7* gene is present in only one copy and was successfully deleted from the *Fol* genome (Supporting Information Fig. S2B). The mutant was also complemented using the wild‐type *FolVAM7* gene with a GFP‐tag at the N‐terminus, as this construct restored all functions (Supporting Information Fig. S3).

### *FolVam7 plays important roles in hyphal growth polarity and asexual development*

**Figure 1:**
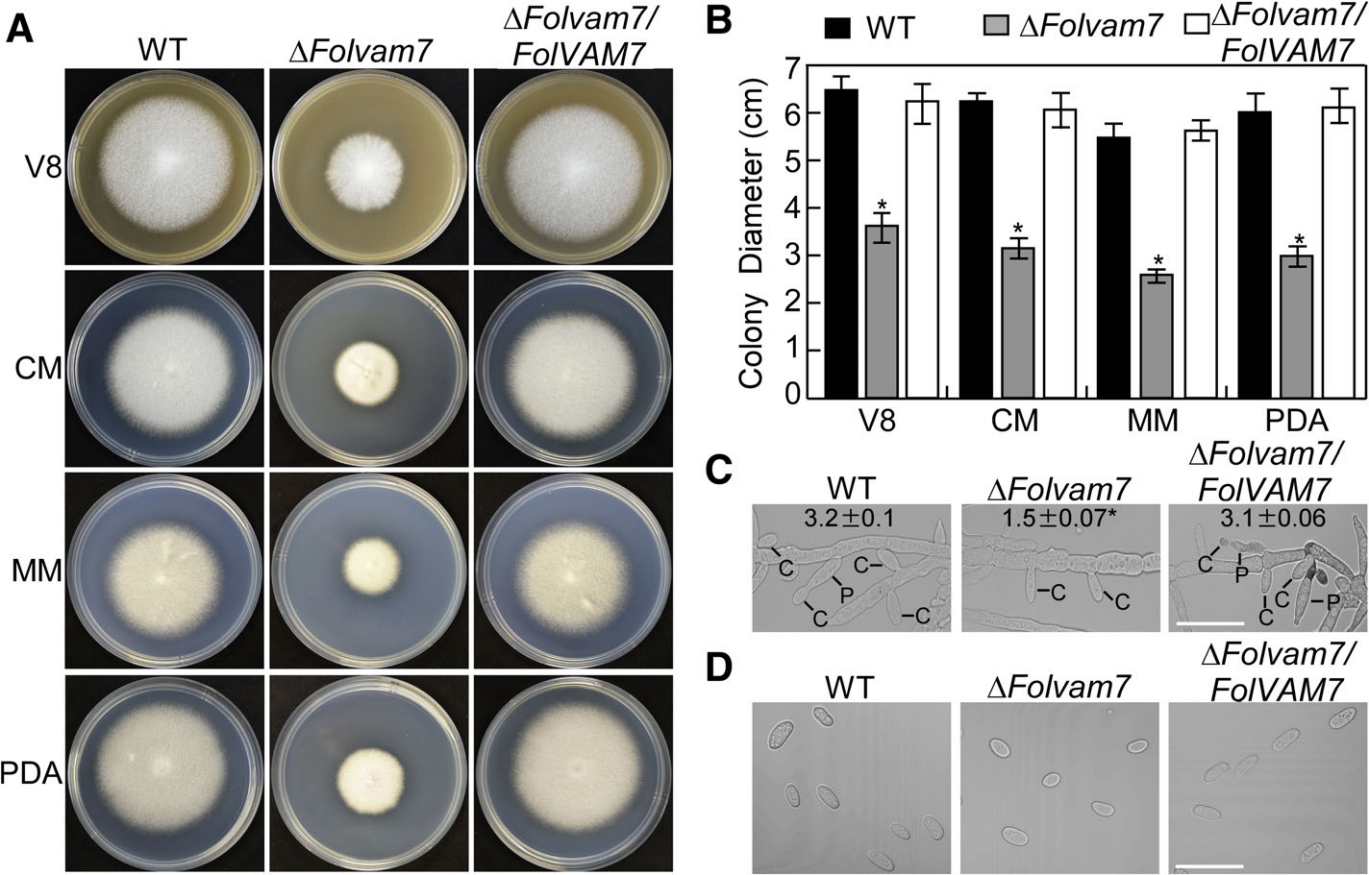
FolVam7 is important for vegetative growth and asexual development. A and B. Colonies of wild‐type, *ΔFolvam7* and the complemented strain were cultured on different media at 28°C for 5 days in the dark. Photographs were taken at 5 days after incubation (dai) using a Nikon digital camera. V8, (V8 juice Medium), complete medium (CM), minimal medium (MM) and potato dextrose medium (PDA). A. Colony images on four media. B. Statistical analysis of the colony diameters in (A), the data represent three replicates. C. Analysis of conidiophore formation. The indicated strains were cultured in carboxymethyl cellulose liquid medium for 5 days and then photographed under differential interference contrast (DIC) microscopy. Bar = 10 μm. C, conidium; P, phialides. D. Conidial morphology of wild‐type *Fol*, *ΔFolvam7* and the complemented strain was examined by DIC microscopy. Bar = 5 μm. Error bars represent the standard deviation and asterisks indicate statistically significant differences relative to wild type (*p* < 0.01). [Color figure can be viewed at http://wileyonlinelibrary.com]

To unravel the cellular function of FolVam7 in *Fol*，we first examined the vegetative growth of the *ΔFolvam7* mutant on V8 juice agar plates at 28°C. After 5 days incubation, the *ΔFolvam7* mutant formed a smaller colony with fewer aerial hyphae compared with the wild‐type and the complemented strain (Fig. 1A). To determine whether this growth defect was medium dependent, we examined the vegetative growth of *ΔFolvam7* on complete medium (CM), minimal medium (MM) and potato dextrose agar (PDA). The results showed that the vegetative growth of *ΔFolvam7* was severely attenuated on all media tested (Fig. 1A and B).

During the disease cycle of tomato wilt, conidia produced by *Fol* mainly infect the host root (Mes *et al*., 1999; Duyvesteijn *et al*., 2005). Therefore, we examined the role of FolVam7 during conidia formation. Microscopic observations showed that conidiophore production by *ΔFolvam7* was decreased by approximately 50% compared with the wild‐type and complemented strains (Fig. 1C). Furthermore, 73% of the conidia produced by the *ΔFolvam7* mutant were smaller than those from the wild type. The average size of conidia from the wild‐type strain was approximately 10.4 μm ± 1.6 μm while that from the *ΔFolvam7* mutant was 6.2 μm ± 1.8 μm (Fig. 1D; data not shown). Taken together, the results from phenotypic analysis suggested that FolVam7 plays a crucial role in vegetative growth, conidiation and conidial morphogenesis in *Fol*.

### *FolVam7 is required for pathogenicity*

**Figure 2:**
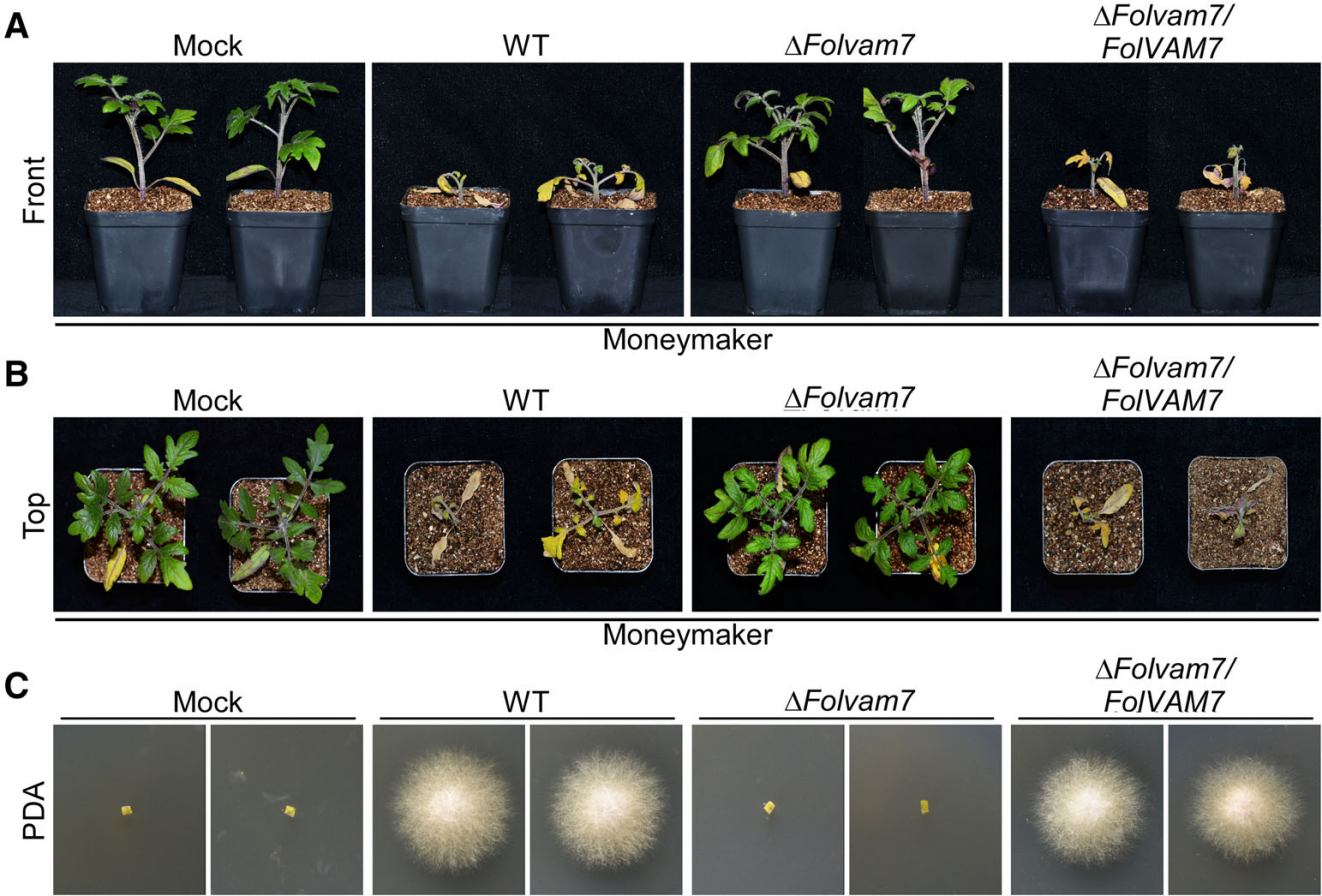
FolVam7 is required for full virulence.A and B. Infection assays performed using the susceptible cultivar Moneymaker. Tomato seedlings (12 days old) were infected using a standard root‐dip method with conidia of wild type, *ΔFolvam7* and the complemented strain. Photographs were taken at 21 days after infection. C. The outgrowth of fungi from tomato stems of plants inoculated with the indicated strains on PDA. [Color figure can be viewed at http://wileyonlinelibrary.com]

To investigate a possible function for FolVam7 in pathogenicity, 12‐day‐old susceptible cultivar Moneymaker seedlings were inoculated with conidial suspensions of each wild type, *ΔFolvam7* and complemented strains. After 21 days, the tomato seedlings treated with *ΔFolvam7* conidial suspensions showed no obvious wilt symptoms, in contrast to plants treated with the wild type or complemented strains (Fig. 2A and B). In order to further understand the *ΔFolvam7* pathogenesis defect, fungal recovery assays were performed by placing stem sections from infected plants on PDA plates. As expected, infected stem sections from the Δ*Folvam7* mutant exhibited little fungal growth (Fig. 2C). In contrast, stem sections from wild type and the complemented strain supported good fungal growth (Fig. 2C). The fungal biomass quantification results further confirmed these observations, with much greater levels in wild type and complemented strains than the Δ*Folvam7* mutant (Supporting Information Fig. S4). These results indicated that FolVam7 is required for full virulence in *Fol*.

**Figure 3:**
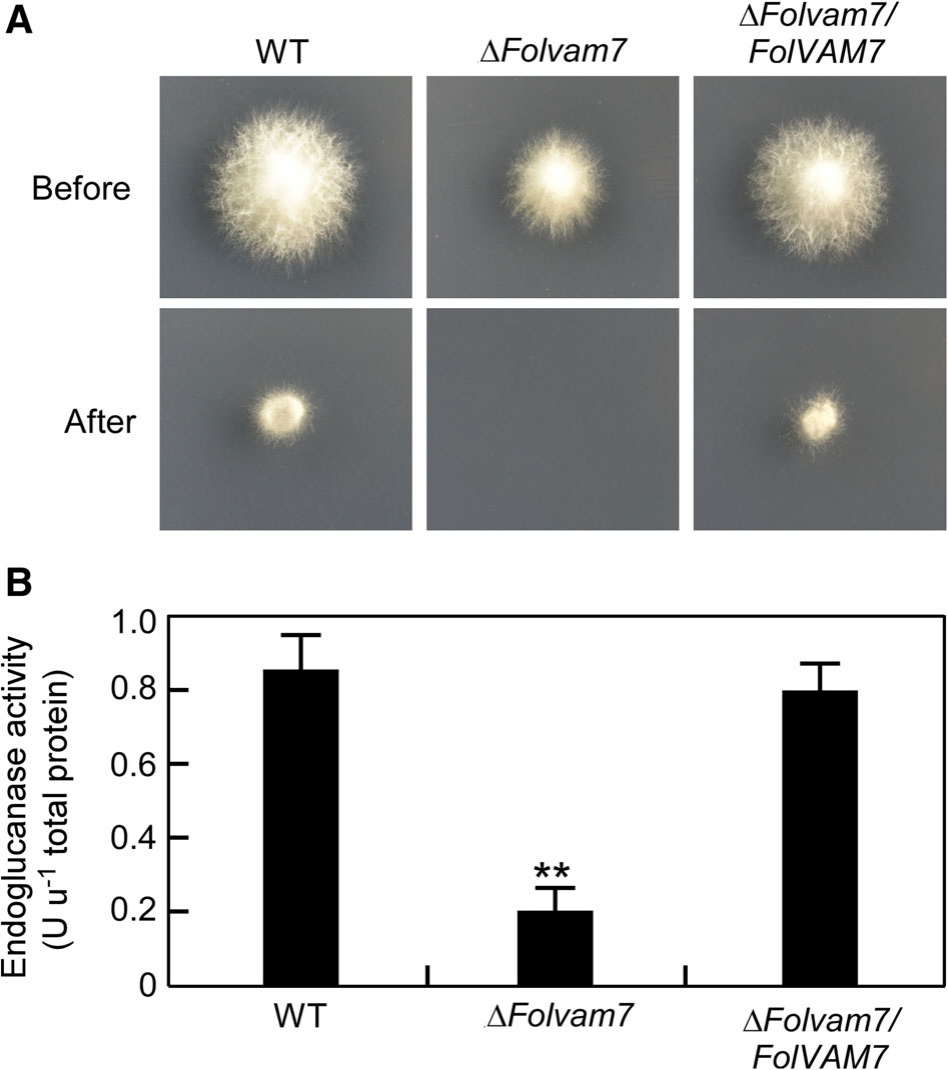
FolVam7 is important for endoglucanase activity. A. Penetration of cellophane by fungal hyphae. Strains were inoculated on the top of a cellophane membrane placed on a minimal medium plate and then incubated at 28°C in the dark. After 2 days of incubation, the cellophane membranes and the fungal colony were removed, and the plates were incubated for an additional day and photographed. Fungal hyphae that have penetrated the cellophane are able to form a colony on the exposed agar surface. B. Measurements of endoglucanase activity. The endoglucanase activity in mycelia was measured spectrophotometrically. One unit of enzyme activity is defined as 1 nmol min^−1^ of reducing sugars released from the substrate. Error bars represent the standard deviation and asterisks indicate statistically significant differences relative to wild type *Fol* (*p* < 0.01). [Color figure can be viewed at http://wileyonlinelibrary.com]

To explore whether a defect in fungal penetration contributed to the *ΔFolvam7* phenotype during infection, we performed a cellophane penetration assay as described previously (Gu *et al*., 2015; Li *et al*., 2018). The results showed that the *ΔFolvam7* strain failed to penetrate the cellophane; conversely, both the wild type and complemented strains successfully penetrated the cellophane (Fig. 3A). We next investigated whether a change the endoglucanase activity might explain the decreased ability of the *ΔFolvam7* strain to penetrate the cellophane (Jenczmionka *et al*., 2003). The assay results demonstrated that the endoglucanase activity is decreased by 80% in the *ΔFolvam7* mutant compared with the wild type (Fig. 3B). Taken together, these data suggested that the reduced endoglucanase activity contributes to the lessened fungal penetration and decreased pathogenicity of the *ΔFolvam7* mutant on tomato.

### *FolVam7 is localized in vesicles and the vacuolar membrane*

**Figure 4:**
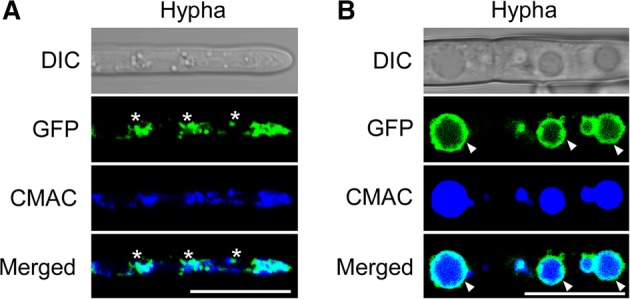
FolVam7 localizes to vesicles and vacuolar membranes.A and B. Apical (A) or basal (B) hyphae of GFP‐FolVam7 strain were incubated on liquid medium for 24 h. CMAC (7‐amino‐4‐chloromethylcoumarin) staining of vacuole was performed at the 37°C for 30 min. Photographs were examined under differential interference contrast (DIC) or epifluorescence microscopy. The merged panels showed the FolVam7 localizes to vesicles and the vacuolar membrane. Asterisk show the vesicle and arrowheads show the vacuole membrane. Bar = 10 μm. [Color figure can be viewed at http://wileyonlinelibrary.com]

As noted earlier, FolVam7 shares high amino acid sequence conservation with other fungal Vam7 proteins. Therefore, we postulated that FolVam7 likely exhibits the conserved cellular functions in vesicle trafficking. We first investigated the intracellular localization pattern of FolVam7 by constructing a strain with a GFP‐tag at the N‐terminus of FolVam7 (GFP‐FolVam7) in the *ΔFolvam7* mutant genetic background. As mentioned previously, this construct rescued *ΔFolvam7* phenotypes. In the *ΔFolvam7/FolVAM7* strain, we observed many punctate fluorescent structures in the apical region and ring‐like structures in the basal region of hyphae respectively (Fig. 4A and B). A previous study reported that the HOPS complex subunit FgVps41 localizes in endosomes and vacuolar membranes in *F. graminearum* (Li *et al*., 2018). We further investigated the ring‐like location in *Fol* by treating cells with 7‐amino‐4‐chloromethylcoumarin (CMAC), a fluorescent dye that stains the interior of vacuoles. We found that the majority of the GFP‐FolVam7 signal partially co‐localized with CMAC at the apical region of the hyphae (Fig. 4A) and in the ring‐like structures (vacuolar membranes) surrounding the vacuole interior at the basal region of hyphae (Fig. 4B). These results demonstrated that FolVam7 localizes to vesicles and the vacuolar membrane.

### *The PX and SNARE domains of FolVam7 are indispensable for normal cellular localization and biological functions*

**Figure 5:**
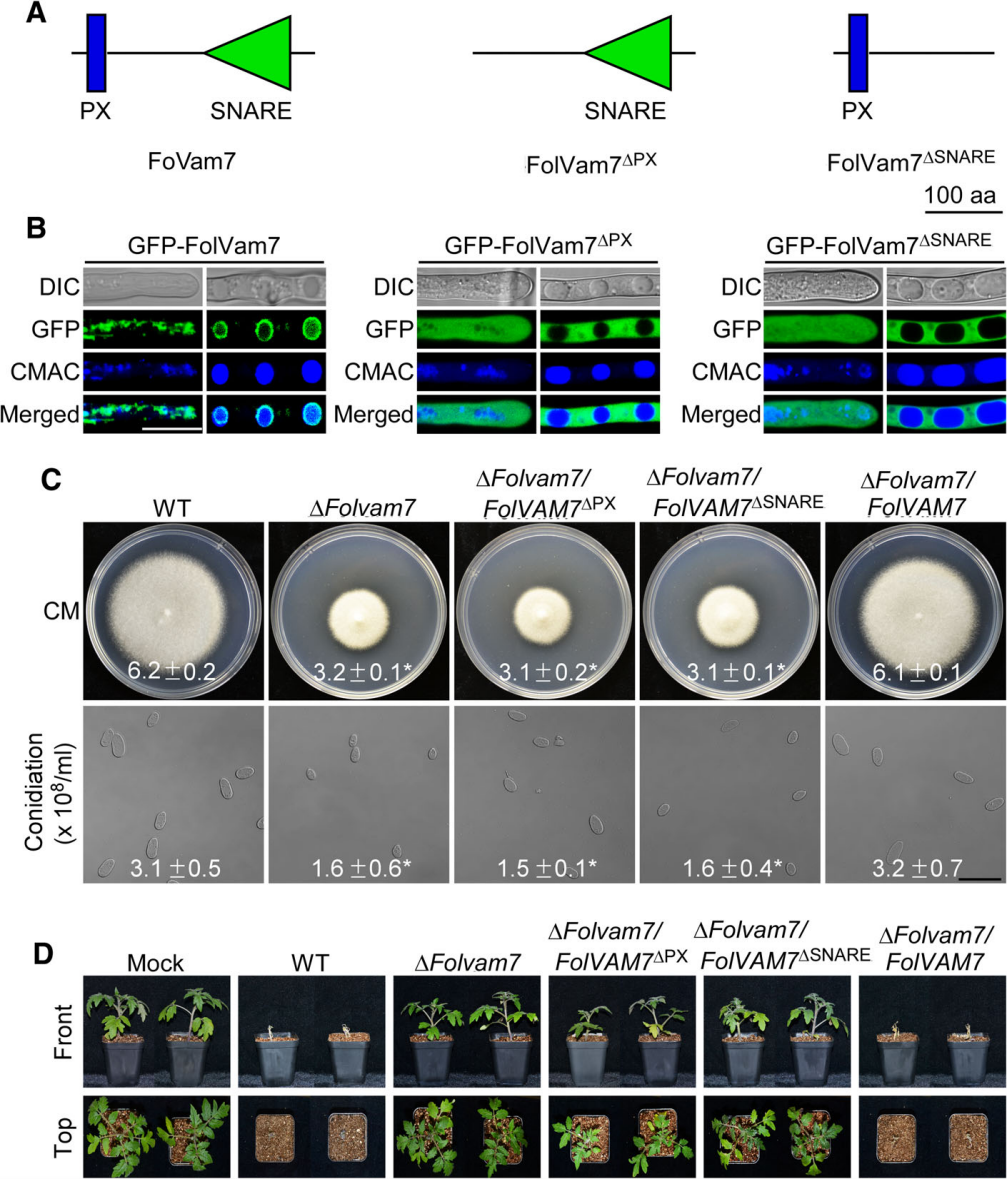
Functional analysis of the PX motif and SNARE domain of FolVam7. A. Schematic showing domain deletions in FolVam7. B. Subcellular localization of the mutated FolVam7 proteins. Hyphae were imaged as described in Fig. 4. C and D. Growth and conidiation assays were performed as described in legend to Fig. 1. Moneymaker tomato plants were infected with the control and domain deletion strains as described in the legend to Fig. 2 and photographed at 21 dai. Bar = 10 μm. ± represents SD and asterisks indicate statistically significant differences (*p* < 0.01). [Color figure can be viewed at http://wileyonlinelibrary.com]

To investigate the importance of each domain (PX and SNARE) of FolVam7 for cellular localization and biological function, we generated FolVam7^*Δ*PX^ and FolVam7^*Δ*SNARE^ deletion domain constructs with a GFP‐tag at the N‐terminus and transformed each construct into the *ΔFolvam7* mutant (Fig. 5A). Both GFP‐FolVam7^*Δ*PX^ and GFP‐ FolVam7^*Δ* SNARE^ failed to be localized in vesicles and vacuolar membrane structures, instead, they were distributed throughout the cytoplasm (Fig. 5B). These results suggested that the PX motif and SNARE domains are crucial for the proper localization of FolVam7 in *Fol*. Additionally, assays of vegetative growth and conidiation indicated that both the PX motif and SNARE domain‐truncated strains displayed phenotypes similar to Δ*Folvam7* (Fig. 5C). Finally, host infection experiments showed that the PX motif and SNARE domain were required for the pathogenicity of *Fol* in the Moneymaker cultivar (Fig. 5D). Therefore, the PX motif and SNARE domains of FolVam7 are indispensable for normal subcellular localization and for the biological functions of FolVam7.

### *FolVam7 is involved in endocytosis and vacuole fusion*

**Figure 6:**
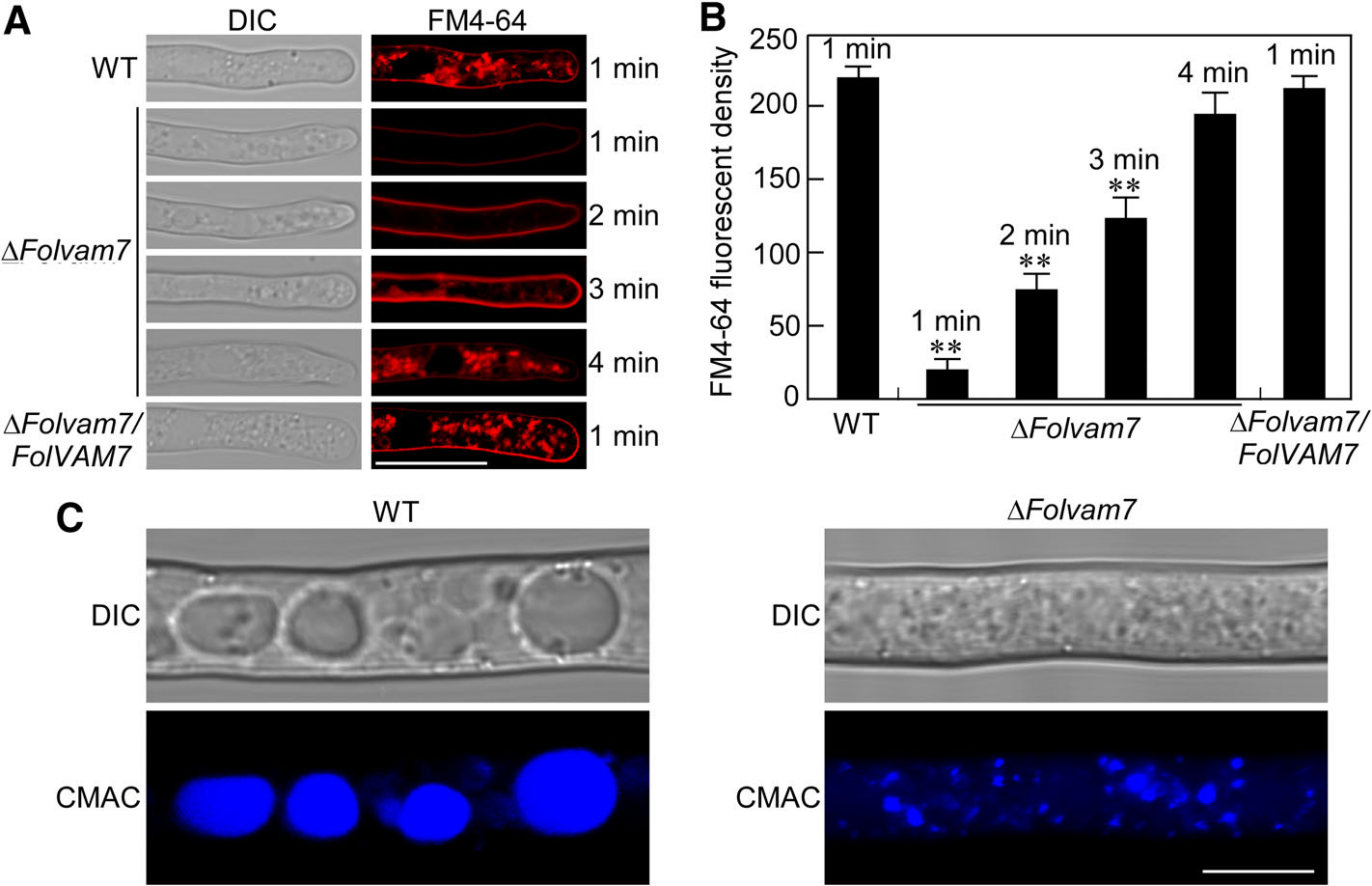
Examination of endocytosis and vacuole fusion in Δ*Folvam7*. A. Hyphae of indicated strains were cultured in liquid CM for 24 h and stained using FM4‐64. Samples were examined using DIC and fluorescence microscopy at different times. Bar = 10 μm. B. Quantitation of FM4‐64 fluorescence. See the Materials and Methods for the detailed procedure. Asterisks indicate statistically significant differences relative to wild type after 1 min of exposure (*p* < 0.01). C. Hyphae of wild‐type and *ΔFolvam7* strains were stained using CMAC and observed by DIC and epifluorescence microscopy. Bar = 10 μm. [Color figure can be viewed at http://wileyonlinelibrary.com]

The localization of FolVam7 in vesicles suggested that it might function during endocytosis. A previous study reported that the FgVam7 protein is localized in vacuoles and involved in endocytosis in *F. graminearum* (Zhang *et al*., 2016). We investigated endocytosis in the Δ*Folvam7* mutant using FM4‐64, a fluorescent dye that stains phospholipids and has been used to track endocytosis in fungi (Fischer‐Parton *et al*., 2000; Naramoto *et al*., 2010; Qi *et al*., 2016). The results showed that wild type and complemented strains took up the FM4‐64 dye within 1 min after exposure (Fig. 6A and B). Fluorescence was detected on the plasma membrane and in numerous endomembrane compartments. In contrast, only a weak fluorescent signal on the plasma membrane was observed for the *ΔFolvam7* mutant after 1‐min exposure, and endomembrane compartments did not exhibit fluorescence until 4 min of incubation (Fig. 6A and B). The results suggested that endocytosis was delayed in the mutant. We further examined the vacuoles by CMAC staining and observed small and fragmented vacuoles in Δ*Folvam7*. Small and fragmented vacuoles were observed in Δ*Folvam7* compared with normal intact vacuoles in the wild‐type strain (Fig. 6C). These findings supported a role for FolVam7 during endocytosis and vacuole fusion in *Fol*.

### *FolVam7 is required for a normal response to various stressors*

**Figure 7:**
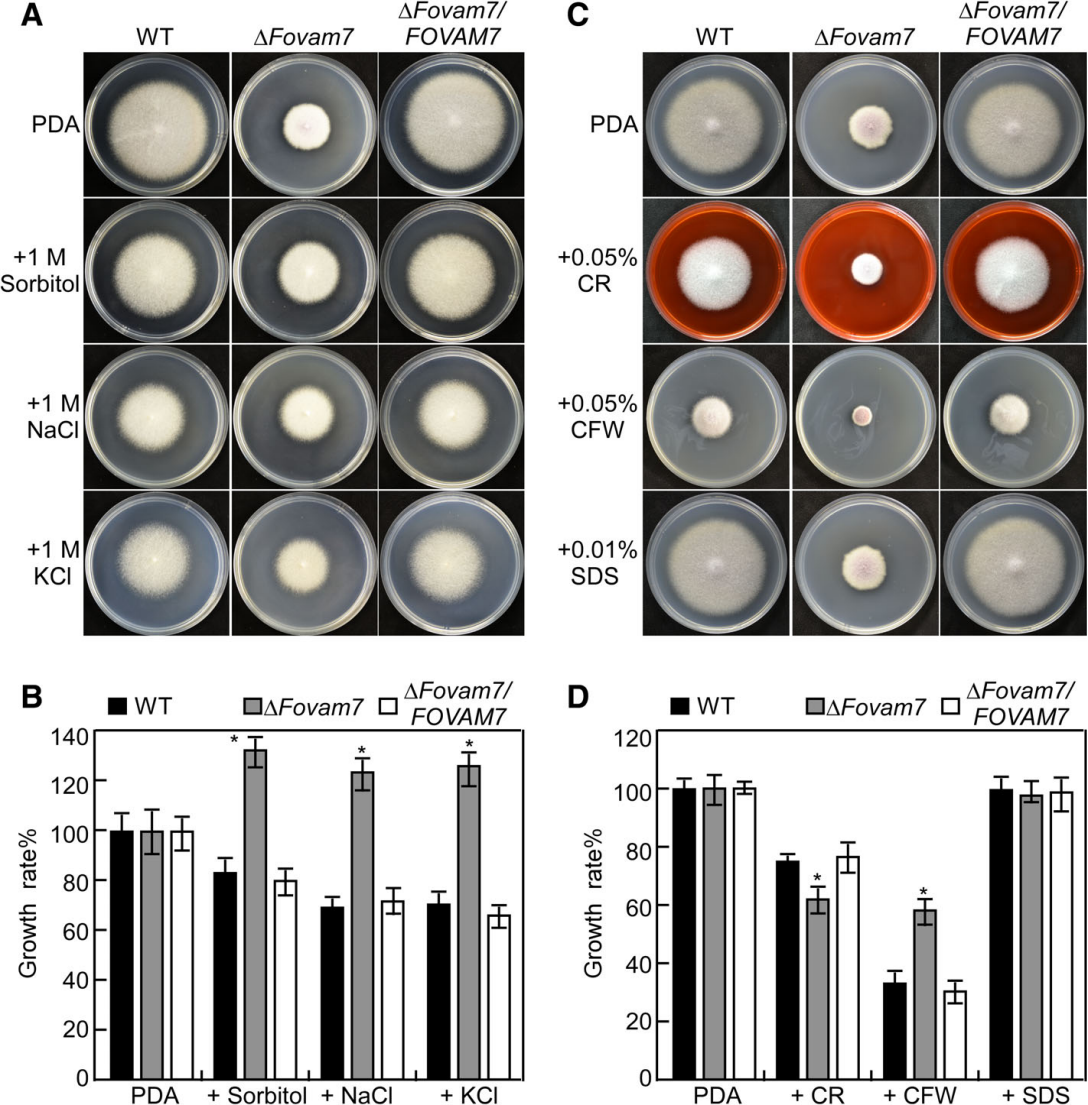
Defects of the Δ*Folvam7* mutant in response to various stressors. A. Wild‐type *Fol4287*, *ΔFolvam7* mutant and the complemented strain were inoculated on CM medium plates alone or supplemented with hyperosmotic stressors sorbitol (1 M), sodium chloride (NaCl, 1 M) or potassium chloride (KCl, 1 M). C. The indicated strains were cultured on plates containing cell wall antagonists Calcofluor white (CFW; 0.05%), Congo red CR (CR; 0.05%) or Sodium dodecyl sulfate (SDS; 0.01%). B and D. The growth rates (artificially set to 100% on CM plates) of the indicated strains under various stress conditions. Error bars represent the standard deviation from three independent experiments and asterisks indicate statistically significant differences relative to wild type (*p* < 0.01). CM, complete medium. [Color figure can be viewed at http://wileyonlinelibrary.com]

Because the Δ*Folvam7* mutant was impaired in vacuole fusion and the timing of endocytosis, we speculated that these defects might influence vesicular transport and the response to various stressors. We therefore examined the sensitivity of the Δ*Folvam7* mutant on CM plates supplemented with NaCl, KCl, sorbitol (osmotic stress), or the cell wall stressors Congo red (CR), Calcofluor white (CFW) and sodium dodecylsulfate (SDS). After 5 days incubation, osmotic stress partially relieved the Δ*Folvam7* mutant growth defect on plates containing 1 M NaCl, 1 M KCl or 1 M sorbitol (Fig. 7A and B). In contrast, the mutant lacking *FolVam7* exhibited heightened sensitivity during growth on plates containing 0.05% CFW or 0.05% CR (Fig. 7C and D). No differences between the three strains were observed on the plates containing 0.01% SDS (Fig. 7C and D). These findings indicated that FolVam7 may have a role in the maintenance of cell wall integrity and osmoregulation.

## Discussion

The transport of various cellular materials is required for the development of eukaryotic organisms, and this processes is mainly mediated by vesicle trafficking. Despite its importance, studies on vesicle trafficking in filamentous fungi are limited. Here, we focused on SNARE proteins, which are important in vesicle trafficking in all eukaryotes studied to date. We identified and functionally characterized the v‐SNARE protein Vam7 in *F. oxysporum* f. sp. *lycopersici* and demonstrated that FolVam7‐mediated vesicle trafficking promotes vegetative growth, conidiogenesis and pathogenicity. This study has yielded new information on regulatory mechanism that is essential for infection‐related morphogenesis and pathogenicity of tomato wilt disease.

The Vam7 protein, as well as many other SNARE proteins, is involved in vesicle trafficking and is localized in vesicles in *M. oryzae* and *F. graminearum* (Sato *et al*., 1998; Dou *et al*., 2011; Zhang *et al*., 2016). Our results are consistent with previous observations and showed that FolVam7 is localized in vesicles at the apical region of the hyphae and vacuole membranes at the basal region of the hyphae. In *S. cerevisiae* and *F. graminearum*, the PX motif and SNARE domains are required for Vam7 function. Moreover, the PX motif of FgVam7 is required for correct localization (Sato *et al*., 1998; Lievens *et al*., 2009; Zhang *et al*., 2016). Our data demonstrate that both the PX motif and SNARE domains are important for the localization and function of FolVam7 in *Fol*. From these observations, we deduced that the SNARE domain of FolVam7 may have a different regulatory mechanism than that in these other fungi.

Endocytosis is a biological process in which extracellular substances are internalized in vesicles by invagination of the plasma membrane (Mellman, 1996; Qi *et al*., 2016). In eukaryotic organisms, endocytosis mediates transportation of proteins and lipids, absorption of nutrients, maintenance of cell polarity and cellular signal transduction (Steinberg and Fuchs, 2004; Higuchi *et al*., 2009; Hao *et al*., 2016). In the pathogenic fungus *M. oryzae*, MoSec22 and MoSyn8 are crucial for hyphal growth and pathogenesis by regulating vesicle trafficking and endocytosis (Houterman *et al*., 2009; Qi *et al*., 2016). FgVam7 mediates endocytosis and vacuole assembly, which are essential for development and virulence in *F. graminearum* (Zhang *et al*., 2016). We found that FolVam7 is involved in development, conidial morphogenesis, pathogenicity and endocytosis of *Fol* and FolVam7 is localized on vesicle and vacuolar membranes. Taken together, our results supported the hypothesis that disruption of FolVam7‐mediated cellular transport leads to defects in development and pathogenicity in *Fol*.

The vacuole is a dynamic structure, and the number and size of vacuole have been found to be associated with different extracellular conditions (Li and Kane *et al*., 2009; Izawa *et al*., 2010). The vacuole fragmentation readjusts the surface‐to‐volume ratio and hence allows reestablishment of tension of the vacuolar boundary membrane (Zieger and Mayer *et al*., 2012). In our study, we found that the Δ*Folvam7* mutant exhibited heightened sensitivity to cell wall stress but reduced sensitivity to osmotic stress. As the Δ*Folvam7* mutant showed a defect in vacuole fusion, it is reasonable to speculate that it will display an altered response to various environmental stresses. In addition, FgVam7 is not involved in conidial morphology (Zhang *et al*., 2016), whereas the FolVam7 is important for conidial morphology in *Fol*. The discrepancies indicate that Vam7 proteins have distinct roles even among various species within the *Fusarium* genus.

In summary, our results suggested that the SNARE protein FolVam7 mediates vesicle trafficking, thus causing pleiotropic effects on polar hyphal growth, asexual development, endocytosis and pathogenesis in *F. oxysporum* f. sp. *lycopersici*. Our work has provided insights into the role of FolVam7 in promoting the morphological and infectious development of *Fol*. Future research is needed to identify the cargo packaged in vesicles and to further understand the role of vesicle trafficking mediated by FolVam7 in the physiological and pathological life cycle of *Fol*. Results from such studies will provide new potential targets for developing sustainable control strategies for tomato wilt disease.

## Experimental procedures

### *Fungal strains and culture conditions*

The wild‐type *Fol* strain used for transformation experiments is FGSC9935 (also referred to as *Fol* 4287 or NRRL 34936) (Ouyang *et al*., 2014; Ji *et al*., 2018; Zhao *et al*., 2018). All strains were maintained on potato dextrose agar (PDA) medium (Zhang *et al*., 2016). Vegetative growth assays were performed on CM, MM and PDA at 28°C for 5 days in dark. For conidiation, strains were cultured in liquid carboxymethylcellulose (CMC) medium and assayed as described previously with minor modifications (Zhang *et al*., 2016). For stress assay, strains were cultured on CM with different concentration of NaCl, KCl, sorbitol, CFW, SDS and Congo red and incubated at 28°C for 5 days in dark. Liquid CM was used to prepare vegetative mycelia for the extraction of genomic DNA and total RNA. Protoplasts prepared from 12 h germlings were used for polyethylene glycol (PEG)‐mediated transformation (Zhou *et al*., 2011; Zhang, HF *et al*., 2016). For transformation, hygromycin B (Solarbio, Beijing, China) and geneticin (MP Biochemicals, Santa Ana, CA) were added at a final concentration of 300 and 400 μg ml^−1^ respectively. For stress assays, all indicated plates were incubated in dark at 28°C for 5 days. The growth rates were scaled by colony diameter and the strain on medium without stressor was used as a control. All experiments were repeated for three times, and for each treatment, 10 plates were applied for replicates.

### *Construction of the FolVAM7 knock out, domain deletion mutants and complemented strain*

In order to analyse the biological function of the *FolVAM7* gene, we constructed a targeted gene replacement vector using the split‐marker approach (Catlett *et al*., 2003). The upstream flanking sequence, downstream flanking sequence and *HPH* cassette were amplified using primers in polymerase chain reactions (PCRs). The PCR products were purified and then transformed into protoplasts of the wild‐type strain as previously described (Zhang *et al*., 2016; Li *et al*., 2017). Subsequently, the hygromycin‐resistant transformants were screened by PCR and further confirmed by Southern blot analysis. For construction of the complementation construct, we amplified a fragment containing the entire gene and GFP sequence using primers and co‐transformed with *Xho* I‐digested pYF11 (confers geneticin resistance) plasmid into yeast, using the yeast gap repair approach to obtain plasmid *GFP‐FolVAM7* (Zhou *et al*., 2011). The complementation construct was then transformed into protoplasta of Δ*Folvam7*. For domain deletion construct generation, primers were designed for splicing overlap extension (SOE)‐PCR and used in PCRs. The transformants were then screened by phenotypic characterization and detection of a GFP signal during fluorescence microscopy. All primers used in this section are listed in the Supporting Information Table S1.

### *Plant infection assays*

*Solanum lycopersicum* cv. C32 (Moneymaker, susceptible *i‐2*) (Kroon and Elgersma *et al*., 1993) and OT364 (Motelle, resistant *I‐2*) (Mes *et al*., 1999) tomato cultivars were employed for infection. Seedlings (12 days old) were infected using a standard root‐dip inoculation method (Wellman *et al*., 1939; Mes *et al*., 1999). Conidia were harvested from 4‐day‐old CMC cultures, followed by washing with sterile water three times and adjusted to a concentration of 5 × 10^6^ conidia/mL. Roots of uprooted seedlings were inoculated using the conidial suspension (treatment) and water (mock) for 30 min respectively. Treated seedlings were then planted in a container with potting soil. Inoculated plants were grown at 28°C, 55%–65% relative humidity and 16‐h light. The plants were examined for typical wilting symptoms after 3 weeks.

Fungal recovery assays were performed as previously described (Fradin *et al*., 2009). Three weeks after inoculation with indicated strains, a stem section immediately above the cotyledons was taken and surface sterilized for 15 min in 70% ethanol, followed by 15 min in 10% hypochlorite and then rinsed three times with sterile water. The sterilized stem was sliced into 2 mm × 2 mm sections. In total, for each plant, 10 slices were transferred onto potato dextrose agar and cultured at 28°C for 2 days in the dark. The average disease index and mean weight of the plant part above the cotyledons were determined as described previously (Rep *et al*., 2004; Ma *et al*., 2015).

### *Endoglucanase activity assay*

In order to assure a comparable physiological status and biomass of the inoculum, the cells were precultivated in CM medium as described previously (Jenczmionka and Schafer, 2005). The conidia of the wild type, Δ*Folvam7* mutant and complemented strains were pre‐cultivated in 100 ml liquid CM containing 1% glucose, 0.05% yeast extract and 1× yeast nitrogen base for 4 days in dark at 28°C, with shaking at 150 rpm. The resulting mycelia were then recovered from the medium by filtration, washed three times with 100 ml sterile water and used to inoculate 100 ml of induction medium containing mineral salts and trace elements. As sole carbon sources, 0.5% CMC sodium salt and 0.2 mg/ml casein were added individually. Samples of the induced cultures were collected at 24 h after inoculation. The culture supernatant was separated from the mycelia by centrifugation for 10 min at maximum speed, supplemented with 0.02% sodium azide, and used for endoglucanase activity assays (Jenczmionka and Schafer, 2005). Endoglucanase activity was measured using carboxmethylcellulose as sole carbon source during growth of the cultures and as the substrate for the enzyme assay. Using this substrate, endoglucanase activity is specifically assayed (Wood and Mahalingeshwara, 1988). Culture supernatant (30 μl) was added to 270 μl of substrate solution (1% CMC sodium salt, dissolved in 50 mM sodium acetate, pH 5.0) and incubated at 37°C. The concentration of reducing sugars was determined using a spectrophotometric assay (Waffenschmidt and Jaenicke, 1987; Gu *et al*., 2015). Calibration curves were made from standard glucose solutions. One unit of enzymatic activity is defined as 1 nmol min^−1^ reducing sugars released from the substrate.

### *RNA isolation and quantitative real‐time PCR*

Total RNA was isolated from roots using the TRIzol LS reagent (Invitrogen) according to the manufacturer's recommendations, and subsequently further purified using RNeasy Mini spin columns (Qiagen). Samples containing 1 μg of total RNA were used for the first strand cDNA synthesis using HiS cript II Reverse Transcriptase (Vazyme Biotech Co., Nanjing, China) following the manufacturer's instructions. The RT2 PCR Real‐Time SYBR Green/ROX PCR master mix (TaKaRa, Dalian, China) was used for qRT‐PCR analysis. Relative quantification of each transcript was calculated using the 2^‐ΔΔCT^ method as previously described (Livak and Schmittgen, 2001). For each experiment, qRT‐PCR assays were repeated three times using independent biological replicates. All primers used in this section were listed in the Supporting Information Table S1.

### *Light microscopy studies*

To examine endocytosis, strains were cultured in liquid CM medium. After growing at 28°C for 24 h, the hyphae were stained with N‐(3‐triethylammoniumpropyl)‐4‐(p‐diethylamino‐phenyl‐hexatrienyl) pyridinium dibromide (FM4‐64) (Molecular Probes, Eugene, OR, USA). This dye is used for staining the Spitzenkorper, plasma membrane, septum, early endosomes, late endosomes and vacuolar membranes, as well as for the examination of endocytosis, as described previously (Fischer‐Parton *et al*., 2000). Photographs were taken using a Zeiss LSM 710 confocal microscope with a 63/1.2 NA C‐Apochromat oil immersion objective (Zeiss, Oberkochen, Germany). The relative fluorescent density was analysed using Image‐pro Plus (Media Cybernetics, Shanghai, China). 7‐amino‐4‐chloromethylcoumarin (Sigma‐Aldrich) (10 mM stock solution in dimethyl sulfoxide) was used for vacuole staining, as previously described (Ohneda *et al*., 2002; Shoji *et al*., 2006). Photographs were taken using the confocal laser scanning microscope, as described earlier.

### *Accession number*

The gene sequences can be found using the following accession number at website (http://eupathdb.org/eupathdb/): *FolVAM7* (FOXG_02319).

### *Statistical analysis*

Each result is presented as the mean ± standard deviation (SD) of at least three replicate measurements. Significant differences between treatments were statistically evaluated by SD and one‐way analysis of variance (ANOVA) using SPSS 2.0 (Chicago, IL, USA). The data for two specific different treatments were compared statistically by ANOVA, followed by Student's T‐test if the ANOVA result was significant at *p* < 0.01 (Chen *et al*., 2014).

## Supporting information


**Fig. S1.** Phylogenetic and structural analysis of FolVam7 and its homologues. A. Phylogenetic analysis of Vam7 homologues from different organisms. A neighbour‐joining tree was constructed using MEGA 5 with 1000 bootstraps. GenBank accession numbers and the corresponding species names are as follows: XP_018235815.1 (*Fusarium oxysporum* f. sp. *lycopersici*, FolVam7); XP_957713.1 (*Neurospora crassa*, NcVam7); XP_002379361 (*Aspergillus flavus*, AfVam7); XP_957713.1 (*Magnaporthe oryzae*, MoVam7); XP_761553.1 (*Ustilago maydis*, UmYup1); NP_011303.1 (*Saccharomyces cerevisiae*, ScVam7). B. Identification of conserved protein domains in FolVam7 and its homologues. PX, PhoX homology domain; SNARE, Soluble N‐ethylmaleimide‐sensitive factor attachment protein receptor; aa, amino acid.

**Fig. S2.** Targeted gene replacement of *FolVAM7* in *Fol*. A. Schematic diagram of the split‐marker gene deletion strategy for *FolVAM7*. B. Results from Southern blot analysis of genomic DNA using gene‐specific or *HPH* probes. DNA from the mutant hybridize only to the *HPH* probe, while that from wild type hybridizes only to the gene‐specific probe.

**Fig. S3.** Schematic diagram showing the GFP‐*FolVAM7* construct.

**Fig. S4** qPCR to determine relative levels of *Fol* in stem sections of inoculated plants. Genomic DNA was isolated from tomato stems of the Moneymaker cultivar infected with various *Fol* strains as described in the Materials and Methods. Quantitative PCRs were performed to evaluate the fungal biomass using primers that amplify the intergenic spacer region of ribosomal 28S. The mean values of three determinations with standard deviations are shown. Asterisks indicate statistically significant differences relative to wild type *Fol* (*p* < 0.01).

**Table S1** Primers used in this study.
