# Supplementary figures and images for: The SNARE protein FolVam7 mediates intracellular trafficking to regulate conidiogenesis and pathogenicity in Fusarium oxysporum f. sp. lycopersici
Source: Environ Microbiol. 2019 Mar 20;21(8):2696–706. doi: 10.1111/1462-2920.14585 (PMC6850041; doi:10.1111/1462-2920.14585)

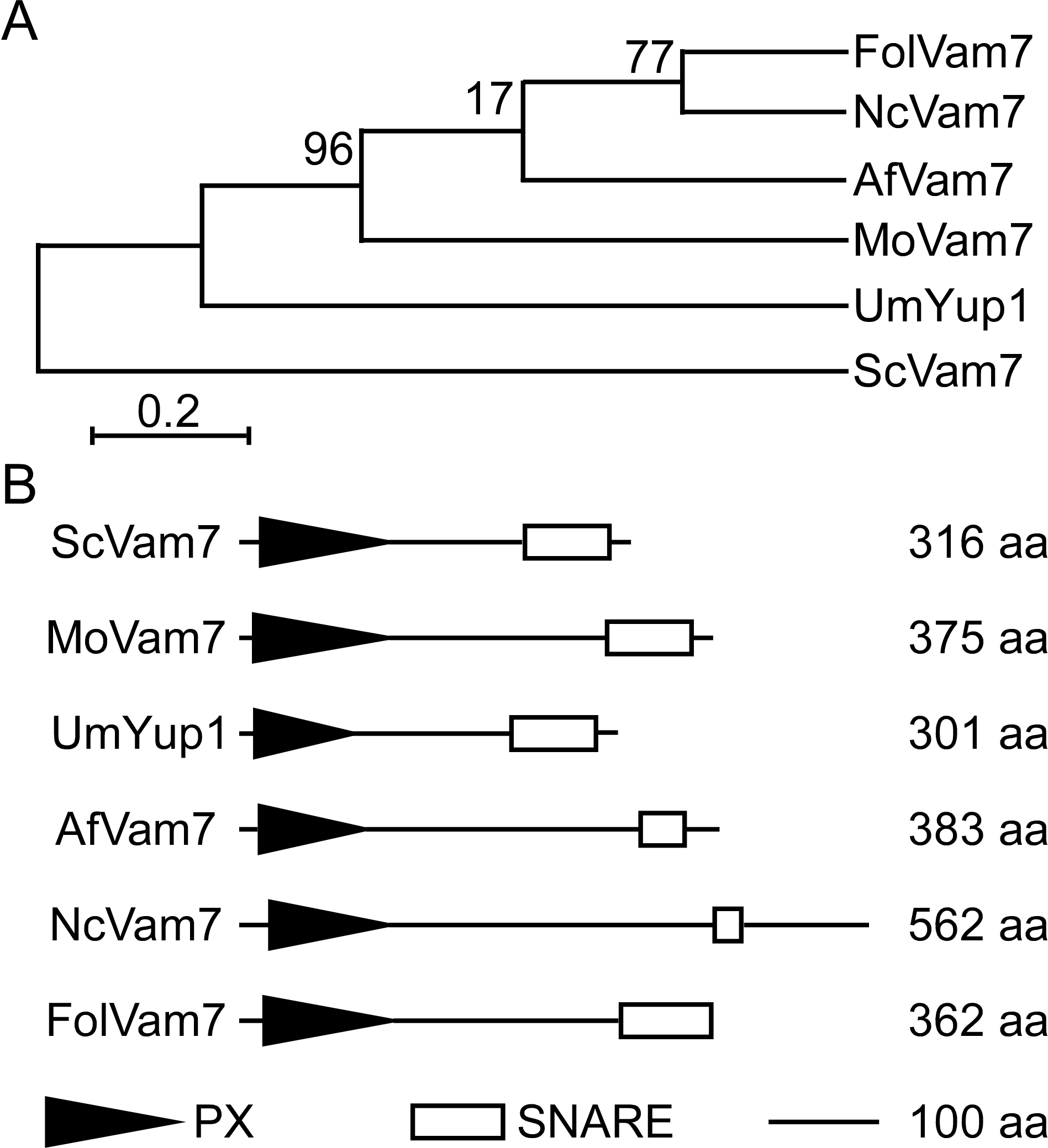

Supplement: Supplementary file 1 — Fig. S1. Phylogenetic and structural analysis of FolVam7 and its homologues. A. Phylogenetic analysis of Vam7 homologues from different organisms. A neighbour‐joining tree was constructed using MEGA 5 with 1000 bootstraps. GenBank accession numbers and the corresponding species names are as follows: XP_018235815.1 (Fusarium oxysporum f. sp. lycopersici, FolVam7); XP_957713.1 (Neurospora crassa, NcVam7); XP_002379361 (Aspergillus flavus, AfVam7); XP_957713.1 (Magnaporthe oryzae, MoVam7); XP_761553.1 (Ustilago maydis, UmYup1); NP_011303.1 (Saccharomyces cerevisiae, ScVam7). B. Identification of conserved protein domains in FolVam7 and its homologues. PX, PhoX homology domain; SNARE, Soluble N‐ethylmaleimide‐sensitive factor attachment protein receptor; aa, amino acid. [file EMI-21-2696-s001.tif]

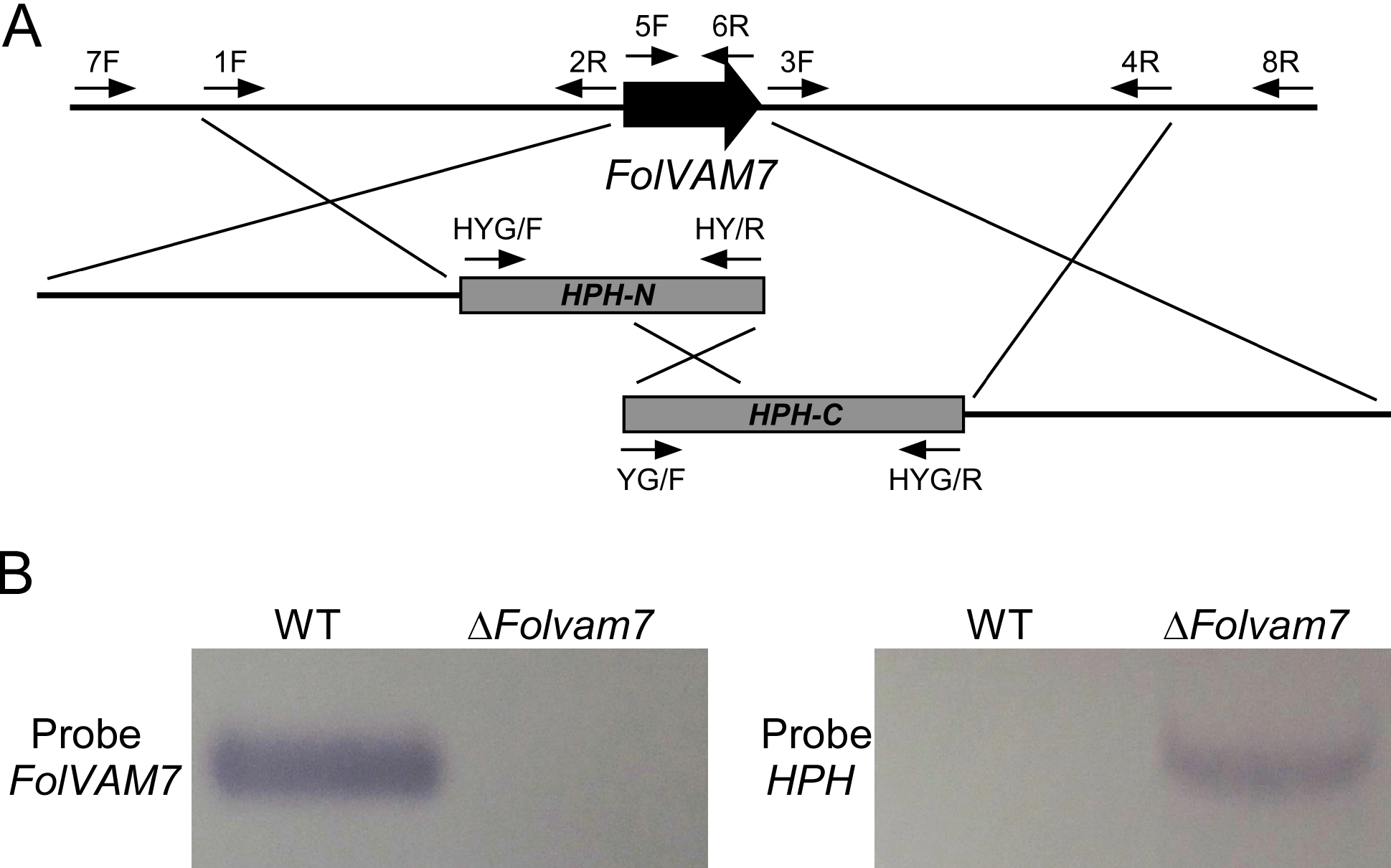

Supplement: Supplementary file 2 — Fig. S2. Targeted gene replacement of FolVAM7 in Fol. A. Schematic diagram of the split‐marker gene deletion strategy for FolVAM7. B. Results from Southern blot analysis of genomic DNA using gene‐specific or HPH probes. DNA from the mutant hybridize only to the HPH probe, while that from wild type hybridizes only to the gene‐specific probe. [file EMI-21-2696-s002.tif]

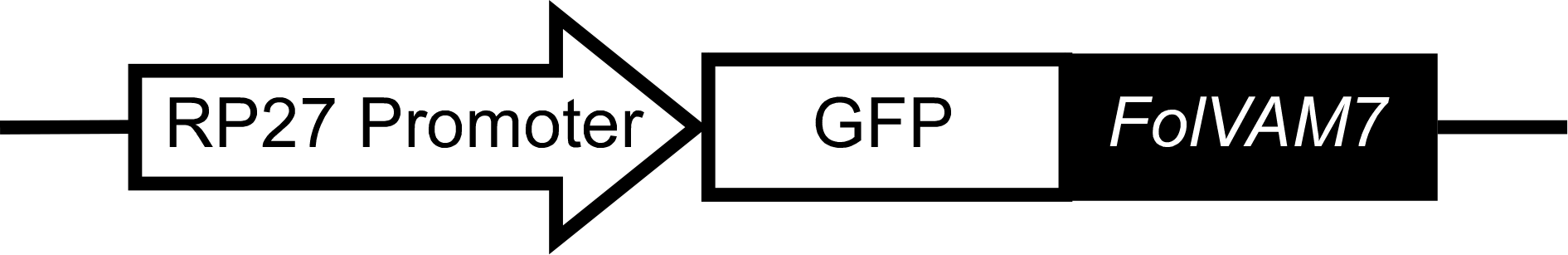

Supplement: Supplementary file 3 — Fig. S3. Schematic diagram showing the GFP‐FolVAM7 construct. [file EMI-21-2696-s003.tif]

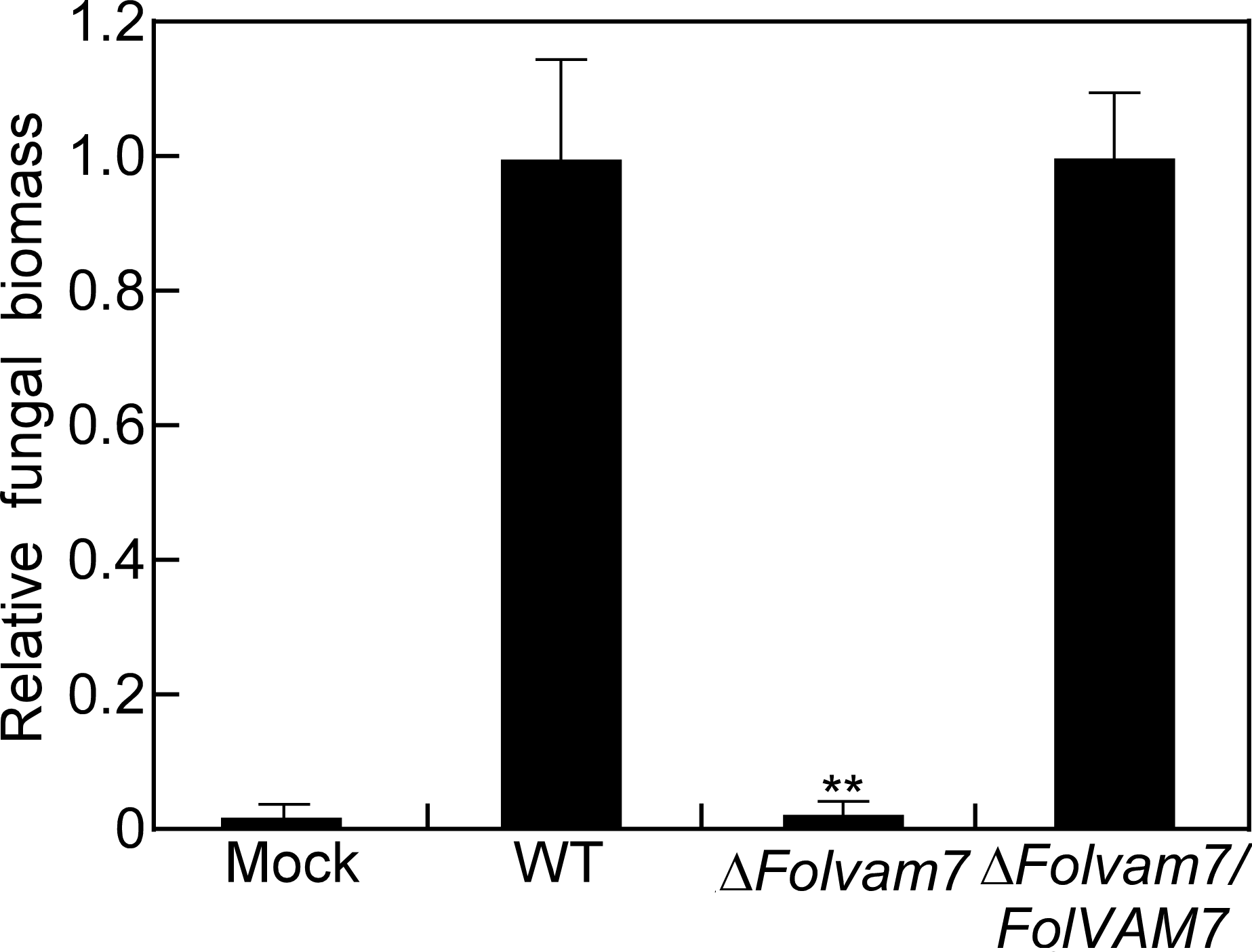

Supplement: Supplementary file 4 — Fig. S4 qPCR to determine relative levels of Fol in stem sections of inoculated plants. Genomic DNA was isolated from tomato stems of the Moneymaker cultivar infected with various Fol strains as described in the Materials and Methods. Quantitative PCRs were performed to evaluate the fungal biomass using primers that amplify the intergenic spacer region of ribosomal 28S. The mean values of three determinations with standard deviations are shown. Asterisks indicate statistically significant differences relative to wild type Fol (p < 0.01). [file EMI-21-2696-s004.tif]
