# Supplementary material for: The SNARE protein FolVam7 mediates intracellular trafficking to regulate conidiogenesis and pathogenicity in Fusarium oxysporum f. sp. lycopersici
Source: Environ Microbiol. 2019 Mar 20;21(8):2696–706. doi: 10.1111/1462-2920.14585 (PMC6850041; doi:10.1111/1462-2920.14585)
Supplement: Supplementary file 5 — Table S1 Primers used in this study. [file EMI-21-2696-s005.docx]

Table S1. Primers used in this study.

| **Primer** | **Sequence (5’-3’)** | **Application** |
| --- | --- | --- |
| *FolVAM7*-1F | TTGCCACGAAACTATGAAGTGCT | Amplify *FolVAM7* 5’ flank sequence, for gene knock out |
| *FolVAM7*-2R | TTGACCTCCACTAGCTCCAGCCAAGCCCTTGGTTGTAGTGATGATAATCGG |  |
| *FolVAM7-3F* | CAAAGGAATAGAGTAGATGCCGACCGGCTACCTCTCTTGCTACTGGTA | Amplify *FolVAM7* 3’ flank sequence, for gene knock out |
| *FolVAM7-4R* | AATACAACACTGTTGAACCCAGG |  |
| *FolVAM7-5F* | GAAGTCGACTGTCAACTCAGTCG | Amplify *FolVAM7* gene probe, for southern blot and transformants screen |
| *FolVAM7-6R* | TTGCTGAGGCCTGGCCTTGACTTA |  |
| *FolVAM7*-7F | TGAGATGTAATTTGGAGCCTGGGA | Transformants screen |
| *FolVAM7*-8R | CCGTTGCCTGGCAACCAGAGATCA |  |
| HYG/F | GGCTTGGCTGGAGCTAGTGGAGGTCAA | Amplify *HPH-N* sequence |
| HY/R | GTATTGACCGATTCCTTGCGGTCCGAA |  |
| YG/F | GATGTAGGAGGGCGTGGATATGTCCT | Amplify *HPH-C* sequence |
| HYG/R | CGGTCGGCATCTACTCTATTCCTTTG |  |
| GFP-FolVam7-1F | TTTCGTAGGAACCCAATCTTCAAAATGGTGAGCAAGGGCGAGGAGCTG-3’  GFP | *For GFP-FolVAM7* fusion construct, constitutive promoter |
| GFP- FolVam7-2R | CTTGTACAGCTCGTCCATGCCGAG |  |
| GFP- FolVam7-3F | CTCGGCATGGACGAGCTGTACAAGATGGCACCGCCGCCAGAAATCGC |  |
| GFP- FolVam7-4R | CACCACCCCGGTGAACAGCTCCTCGCCCTTGCTCACTCACATCTTCTTGATCCGGTTG |  |
| GFP-1F | TTTCGTAGGAACCCAATCTTCAAAATGGTGAGCAAGGGCGAGGAG | *For GFP-FolVAM7*^ΔPX^ fusion construct, constitutive promoter |
| GFP-2R | CTTGTACAGCTCGTCCATGCCGAG |  |
| FolVam7^ΔPX^-1F | CTCGGCATGGACGAGCTGTACAAGATGGCACCGCCGCCAAGCTCGAGCACAACCAACTC |  |
| FolVam7^ΔPX^ -2R | CACCACCCCGGTGAACAGCTCCTCGCCCTTGCTCACTCACATCTTCTTGATCCGGTTG |  |
| GFP-1F | TTTCGTAGGAACCCAATCTTCAAAATGGTGAGCAAGGGCGAGGAGCTG | *For GFP-FolVAM7*^ΔSNARE^ fusion construct, constitutive promoter |
| GFP-2R | CTTGTACAGCTCGTCCATGCCGAG |  |
| FolVam7^ΔSNARE^ -1F | CTCGGCATGGACGAGCTGTACAAGATGGCACCGCCGCCAGAAATCGC |  |
| FolVam7^ΔSNARE^ -2R | CACCACCCCGGTGAACAGCTCCTCGCCCTTGCTCACTCACTCCTCCTTTTGCAGCTGCA |  |
| IGS-1F | TGCGATTTGGACGAGATATGTG | *For* determination *Fol* biomass |
| IGS-2R | ATTTGCCTACCCTGTACCTACC |  |
| SlActin-1F | CTCTCAAGTACCCTATTGAGCAT | Internal for analysis Fol biomass |
| SlActin-2R | CAATACCGGTAGTACGACCACT |  |
